# The effect of probiotic supplements on cognitive outcomes and neuroplasticity in elderly ischemic stroke survivors

**DOI:** 10.3389/fneur.2026.1833163

**Published:** 2026-06-22

**Authors:** Xiaohong Zheng, Wei Chen, Kai Hong, Jiaqing Chen, Yuebin Lin, Jiehua Yang

**Affiliations:** 1 Department of Neurology, The Second Affiliated Hospital of Shantou University Medical College, Shantou, Guangdong, China; 2 Department of General Affairs, The Second Affiliated Hospital of Shantou University Medical College, Shantou, Guangdong, China

**Keywords:** cognitive function, elderly survivors, ischemic stroke, motor recovery, nutritional status, probiotic supplementation

## Abstract

**Background:**

This retrospective cohort study investigates the effects of probiotic supplementation on the nutritional, cognitive, and motor functions of elderly survivors recovering from ischemic stroke. Given the high prevalence of nutritional deficits and cognitive impairments in this population, exploring adjunct therapies such as probiotics could provide improved outcomes.

**Methods:**

The study included 223 elderly ischemic stroke survivors treated between March 2021 and February 2024, divided into two groups based on treatment modalities: the Conventional Group (*n =* 117) receiving standard enteral nutrition, and the Supplement Group (*n =* 106) receiving similar nutrition plus probiotics. Data collected included NIH Stroke Scale (NIHSS), nutritional indicators, cognitive assessments (MMSE, HDS, CDR, SES), and motor evaluations (STREAM, mRS, MBI).

**Results:**

Baseline comparisons showed no significant differences between groups. Post-treatment outcomes demonstrated significant improvements in the Supplement Group across several measures. Neurotrophic and nutritional parameters, including BDNF, hemoglobin, albumin, and prealbumin levels, significantly increased (*p* ≤ 0.011). Cognitive function also improved, with higher MMSE and HDS scores and reduced CDR scores (*p* < 0.003). Motor function, evaluated by STREAM, showed enhanced upper and lower limb mobility and basic activities (*p* ≤ 0.014). Further, participants in the Supplement Group experienced better functional recovery with decreased mRS scores and increased MBI scores (*p* < 0.004). The incidence of gastrointestinal adverse events was significantly reduced in the Supplement Group (*p* < 0.001), and correlation analysis underscored positive associations between probiotics and improved outcomes (*p* ≤ 0.003).

**Conclusion:**

Probiotic supplementation as part of nutritional therapy significantly enhances nutritional, cognitive, and motor function recovery in elderly ischemic stroke survivors, while also reducing gastrointestinal adverse events. These findings suggest that incorporating probiotics into recovery protocols post-stroke may facilitate better health outcomes and provide a well-tolerated therapeutic adjunct.

## 1 Introduction

Stroke remains one of the leading causes of disability and death worldwide, with ischemic stroke accounting for approximately 87% of cases (1). The impact of ischemic stroke is particularly profound among the elderly, who are at risk for substantial cognitive decline and reduced neuroplasticity, which complicates recovery and decreases quality of life (2). Despite advances in acute stroke management, effective strategies to enhance neurological recovery and cognitive rehabilitation in this population are still limited and present a major challenge in clinical practice (3).

Recent studies have highlighted the role of the gut-brain axis—a bidirectional communication network between the gut microbiota and the central nervous system (CNS)—as a promising target for therapeutic interventions that may impact neurological health (4). This axis involves neural, endocrine, and immune pathways, including the vagus nerve, microbial metabolites such as short-chain fatty acids (SCFAs), and modulation of systemic inflammation, all of which can influence stroke outcomes. The gut microbiota has been shown to influence neurodevelopment, modulate neurotransmission, and affect cognitive functions, suggesting that modifications in gut flora may hold keys to enhancing recovery following neurological injuries such as stroke (5). This has led to growing interest in the potential of probiotics, which are live microorganisms that confer health benefits to the host when administered in adequate amounts (6).

Probiotics have been found to exert beneficial effects on gastrointestinal health, systemic inflammation, and metabolic functions, all of which could indirectly influence cognitive outcomes (7). The consumption of probiotics has been associated with increased levels of brain-derived neurotrophic factor (BDNF), a critical protein that supports neuronal survival, synaptic plasticity, and memory formation (8). Given the crucial role of BDNF in neuroplasticity and cognition, probiotics may offer a unique avenue to enhance recovery in stroke survivors through pathways that reinforce synaptic and cognitive functions (9).

Moreover, ischemic stroke often leads to a state of dysbiosis, an imbalance in the gut microbial community, which can exacerbate systemic inflammation and contribute to poor recovery outcomes (10). Probiotics may play a corrective role, restoring microbial equilibrium and reducing pro-inflammatory cytokines that can have deleterious effects on the brain (11). This anti-inflammatory action might not only protect against further neuronal damage but also facilitate neuronal repair and regeneration, potentially improving cognitive rehabilitation outcomes (12).

While some clinical trials have investigated the role of probiotics in general populations with neurological conditions, there is a paucity of research specifically targeting elderly individuals recovering from ischemic stroke (13, 14). This demographic is particularly vulnerable due to age-related declines in cognitive function and neuroplasticity, which may hinder recovery (14). Understanding the therapeutic potential of probiotics in this subset could inform personalized management strategies that incorporate dietary modifications to augment rehabilitation efforts.

The current study seeks to fill this gap by exploring the effects of probiotic supplementation on cognitive outcomes and indicators of neuroplasticity in elderly ischemic stroke survivors. We hypothesize that regular intake of probiotics will be associated with improved cognitive scores and enhanced neuroplasticity, as evidenced by increased BDNF levels and improved performance on cognitive assessments commonly used in stroke rehabilitation.

## 2 Materials and methods

### 2.1 Case selection

*Inclusion criteria*: (1) Participants aged over 60 years who are able to cooperate with various treatments and examinations; (2) Individuals who meet the diagnostic criteria (15) for ischemic stroke during the recovery period of the disease; (3) Patients who have been informed and voluntarily agree to participate in this study.

*Exclusion criteria*: (1) Patients with malignant tumors; (2) Individuals with serious internal medicine diseases or other neurological disorders; (3) Patients suffering from neurological disorders or cognitive impairments; (4) Individuals diagnosed with vascular dementia; (5) Patients with severe diseases affecting the heart, liver, kidneys, hematopoietic system, or immune system; (6) Patients who received antibiotic therapy within 4 weeks prior to enrollment; (7) Patients with a history of severe gastrointestinal diseases (e.g., inflammatory bowel disease, chronic diarrhea, gastrointestinal malignancy, or prior gastrointestinal surgery affecting absorption).

This retrospective cohort study included 223 elderly survivors of ischemic stroke who were admitted to our institution between March 2021 and February 2024. The sample size was determined based on a primary outcome of change in MMSE score. Assuming a two-sided significance level of 0.05 and a power of 80%, and anticipating a mean difference of 2.0 points in MMSE with a standard deviation of 3.5, a minimum of 98 patients per group was required. Considering a potential dropout rate of 10%, the target enrollment was set at 108 patients per group. The final sample of 117 in the Conventional Group and 106 in the Supplement Group met this requirement. Data were systematically collected upon patient admission, encompassing general information, nutritional status, cognitive functioning, motor skills, the modified Rankin Scale (mRS) scores, the Modified Barthel Index (MBI) scores, and the occurrence of adverse reactions. To ensure group homogeneity and minimize selection bias, all patients were consecutively enrolled according to the predefined inclusion and exclusion criteria. No additional patients were excluded after initial screening to achieve statistical balance; the non-significant baseline differences shown in Table 1 reflect the success of the matching process based on the selection criteria rather than post-hoc exclusion.

**Table 1:** Comparison of general information between two groups.

Parameters	Conventional group (*n =* 117)	Supplement group (*n =* 106)	*t*/*χ*^2^	*P*
Age (years)	70.5 ± 5.2	70.3 ± 5.1	0.288	0.774
Gender (Male/ Female)	69/48	60/46	0.128	0.72
body mass index (kg/m^2^)	25.2 ± 3.4	25.1 ± 3.3	0.218	0.827
Education level (years)	10.5 ± 3.2	10.4 ± 3.1	0.234	0.815
Hypertension [*n* (%)]	78 (66.7%)	72 (67.9%)	0.04	0.842
Diabetes mellitus [*n* (%)]	35 (30.0%)	31 (29.2%)	0.012	0.913
Marital status [*n* (%)]	92 (78.6%)	83 (78.3%)	0.004	0.952
Employment [*n* (%)]	19 (16.2%)	18 (17.0%)	0.022	0.882
Smoking history [*n* (%)]	42 (35.9%)	38 (35.8%)	0	0.994
Drinking history [*n* (%)]	21 (17.9%)	19 (17.9%)	0	0.996
Previous stroke [*n* (%)]	28 (23.9%)	25 (23.6%)	0.004	0.952
NIHSS score	12.36 ± 4.12	12.68 ± 4.16	0.562	0.574
VFSS score	3.23 ± 1.07	3.17 ± 1.02	0.409	0.683

NIHSS, the national institutes of health stroke scale; VFSS, video fluoroscopic swallowing study.

Given that this retrospective study utilized only de-identified patient data, there is no potential for causing harm or affecting the medical care of the patients involved; therefore, informed consent was waived. This waiver, as well as the study itself, received approval from our institutional ethics review board, ensuring compliance with regulatory and ethical standards for retrospective research.

### 2.2 Grouping and treatment methods

Based on the differing treatment approaches selected by patients, participants were categorized into two groups: the Conventional Group (117 individuals) and the Supplement Group (106 individuals). Both groups received standard treatments, which included intracranial pressure reduction, nerve nourishment, infection control, and the maintenance of water, electrolyte, and acid–base balance.

For the Conventional Group, enteral nutrition support was provided through the administration of a nutrition emulsion (Fresenius Kabi Deutschland GmbH, National Medical Standard J20140077, specification: 500 mL per bottle) via a nasogastric feeding tube. The initial dose was set at 125 mL, which was gradually increased from 125 mL per day to 1,000 mL per day. During the early phase of enteral nutrition support, an appropriate amount of intravenous nutrition was administered to address any caloric deficits.

In contrast, the Supplement Group received enteral nutrition support supplemented with probiotics. This included *Bifidobacterium lactis* triple active bacterial tablets (containing *B. longum, L. acidophilus,* and *E. faecalis*) (Inner Mongolia Shuangqi Pharmaceutical Co., Ltd., national drug approval number S1998004, specification: 0.5 g), Enteral administration via nasogastric tube was chosen because many elderly stroke survivors in the acute or early recovery phase present with dysphagia, impaired consciousness, or poor oral intake, making reliable oral administration difficult. Enteral nutrition ensures accurate dosing, complete delivery of probiotics, and avoidance of oropharyngeal aspiration, thereby enhancing treatment compliance and safety. These strains were selected because they are commonly used in clinical settings for their stability, safety, and documented effects on gut microbiota modulation, anti-inflammatory properties, and potential neuroprotective effects via the gut-brain axis. The specific strains (*B. longum* strain R0175, *L. acidophilus* strain R0052, and *E. faecalis* strain R0026) were selected based on published evidence showing their ability to modulate gut microbiota composition, reduce systemic inflammation, enhance intestinal barrier function, and increase BDNF levels in preclinical and clinical studies. *B. longum R0175, L. acidophilus R0052, and E. faecalis R0026* were originally isolated from healthy human intestinal flora and have been commercially developed for clinical use. *Bifidobacterium lactis* (synonym of *B. longum*) was chosen because it is one of the most widely studied probiotics for neurological conditions, with demonstrated safety in elderly and medically compromised populations. These strains have also demonstrated safety and stability in enteral feeding formulations. The administration and dosage of the enteral nutrition emulsion were identical to those utilized in the Conventional Group. Treatment duration for both groups was 6 weeks.

### 2.3 NIHSS (the national institutes of health stroke scale)

The scale encompasses assessments of several key domains, including consciousness, language, movement, sensation, ataxia, eye movement, and visual field, with total scores ranging from 0 to 42. A higher score indicates a more severe neurological deficit. Specifically, a National Institutes of Health Stroke Scale (NIHSS) score of 4 or less suggests a mild stroke, whereas a score of 21 or above indicates a severe stroke. However, the scale has notable limitations, including its insensitivity to infarct scores related to the posterior circulation and its failure to evaluate cognitive function and gait abnormalities. Notably, the scale demonstrated a Cronbach’s alpha coefficient of 0.6885 (2), reflecting its reliability (16).

### 2.4 VFSS (Video Fluoroscopic Swallowing Study)

The Video Fluoroscopic Swallowing Study (VFSS) has long been regarded as the gold standard for assessing swallowing function (17). In this study, a 200-mg barium solution was mixed with 286 mL of water to create a radiopaque material at a concentration of 60%. Rice flour was then added to produce thin liquids, thick liquids, or solid food textures. Each participant was positioned upright in a fluoroscopy chair, ensuring that the head and neck were aligned in a neutral position while the chest and abdomen were oriented in the true lateral plane.

Participants sequentially consumed the prepared liquids, beginning with a volume of 2 mL, followed by 5 mL and then 10 mL. Fluoroscopy was employed to capture images of the swallowing process from both lateral and anteroposterior perspectives. The fluoroscopic images encompassed the region from the lips anteriorly to the pharyngeal wall posteriorly, and from the soft palate superiorly to the sixth cervical vertebra inferiorly. These images were recorded at a rate of 30 frames per second and were digitally stored on a personal computer for subsequent frame-by-frame analysis by a physiatrist with expertise in VFSS interpretation. The VFSS scores ranged from 0 to 10, with higher scores indicating superior swallowing ability.

### 2.5 Nutritional indicator

A 5 mL sample of venous blood was collected from each patient, fasting prior to 8 a.m. Hemoglobin levels were assessed using a DxH800 blood analyzer (Beckman Coulter, Inc., Brea, CA, USA). The sample was then centrifuged at 3000 rpm for 5 min to obtain the supernatant. Serum albumin and prealbumin levels were measured using an automated biochemical analyzer (Beckman Coulter, Inc., Brea, CA, USA) via immunoturbidimetry. Additionally, BDNF levels were evaluated using an enzyme-linked immunosorbent assay (ELISA), following the manufacturer’s instructions for the kit (ab229395, Abcam, USA).

### 2.6 Cognitive function assessment

1 Mini-Mental State Examination (MMSE)

The Mini-Mental State Examination (MMSE) is a straightforward and widely utilized tool for assessing cognitive function, particularly in the context of dementia screening, both domestically and internationally. This scale evaluates seven cognitive domains: time orientation, location orientation, immediate memory, attention and calculation, delayed memory, language, and visual–spatial skills. It consists of 30 questions, with one point awarded for each correct answer and zero points for incorrect or unclear responses. The total score ranges from 0 to 30 points. It is important to note that test scores are closely associated with the individual’s educational level; the thresholds for normal cognitive function are defined as follows: illiterate individuals scoring above 17 points, primary school graduates above 20 points, and those with secondary education or higher above 24 points. The MMSE attained a Cronbach’s alpha coefficient of 0.78 (18).

2 Hasegawa Dementia Scale (HDS)

The Hasegawa Dementia Scale (HDS) consists of 11 items that assess cognitive abilities across five domains: common sense, memorization, memory, and computational skills. The total possible score is 32.5 points, with scores above 30.2 indicating normal cognitive function. Scores between 30.5 and 22 are categorized as subnormal, scores between 21.5 and 10.5 suggest possible dementia, and scores from 10 to 0 indicate dementia. In practical settings, only those with severe dementia typically score below 10 points. Research indicates that scores on this scale correlate with the level of education—lower educational attainment is associated with lower scores. The HDS achieved a Cronbach’s alpha coefficient of 0.92 (19).

3 Clinical Dementia Rating (CDR)

The Clinical Dementia Rating (CDR) scale categorizes cognitive impairment with scores of 0.5, 1, 2, and 3 representing suspected dementia, mild dementia, moderate dementia, and severe dementia, respectively. Memory (M) is the primary evaluation component, while other items serve as secondary metrics. If at least three secondary items score equivalently to the memory score, then CDR equals the memory score. Conversely, if three or more secondary items are rated higher or lower than the memory score, CDR reflects the majority score of those secondary items. When three secondary items score on one side of M and two on the opposite, CDR equates to M. Specifically, when M equals 0.5, if at least three other items are rated as 1 or higher, CDR equals 1. Notably, when M equals 0.5, CDR cannot be 0; it may only be classified as 0.5 or 1. If M is 0 and CDR is also 0, this can only occur if there is significant impairment (0.5 or higher), at which point CDR equals 0.5. The CDR attained a Cronbach’s alpha coefficient of 0.88 (20).

4 Self-Esteem Scale (SES)

The Self-Esteem Scale (SES) is utilized to evaluate patients’ self-esteem responses during nursing care, with higher scores indicating a greater level of self-esteem. The SES achieved a Cronbach’s alpha coefficient of 0.86 (21).

### 2.7 Stroke rehabilitation assessment of movement (STREAM)

The Stroke Rehabilitation Motor Function Scale (STREAM) is employed to evaluate the motor function of two patient groups before and after treatment. This scale is divided into three sections: upper limb movements, lower limb movements, and basic activities. Both upper and lower limb movements are assessed using a three-level rating system with scores ranging from 0 to 2 points. The basic activities section uses a four-level rating system, with scores ranging from 0 to 3 points. The total score possible is 70 points, where a higher score indicates better motor function. The scale has demonstrated Cronbach’s alpha coefficients exceeding 0.98 (22).

### 2.8 Modified Rankin Scale (mRS)

The mRS is primarily used to measure neurological recovery outcomes in stroke patients. Serving as a therapeutic indicator of functional disability, this ordinal scale ranks levels of recovery, with higher grades reflecting poorer neurological outcomes. The scoring ranges from 0, indicating no symptoms, to 5, denoting severe disability. Additionally, in clinical trials, a score of 6 is often used to represent mortality. This scale has demonstrated strong reliability and validity (23).

### 2.9 Modified Barthel Index (MBI)

The MBI is a widely used tool for evaluating activities of daily living. It assesses 10 domains, including eating, bathing, grooming, dressing, bowel control, urinary control, transferring between bed and chair, walking on flat surfaces, and climbing stairs. Each item is scored on a scale of 0 to 5 points, 0 to 10 points, or 0 to 15 points, yielding a total score that ranges from 0 to 100 points. A higher score indicates improved performance in daily living activities. The scoring categories are as follows: 100 points indicate normal functioning; scores above 60 reflect basic self-care ability; scores between 41 and 59 indicate moderate functional impairment requiring assistance; scores between 21 and 40 indicate severe functional impairment with significant dependence; and scores below 20 signify complete dependence in daily living. The MBI is recognized for its high reliability and validity (24).

### 2.10 Statistical method

Measurement data are presented as means ± standard deviations or as medians with interquartile ranges, depending on normality. Categorical data are expressed as frequencies and percentages. To compare continuous variables between the two groups, unpaired *t*-tests are utilized. Statistical significance is defined as *p* < 0.05. All statistical analyses were performed using SPSS version 19 (SPSS Inc., Chicago, IL, USA) and R software version 3.0.2 (Free Software Foundation, Inc., Boston, MA, USA). This study is a retrospective cohort study and was not prospectively registered. The datasets used and/or analyzed during the current study are available from the corresponding author on reasonable request. This retrospective cohort study was reported in accordance with the STROBE (Strengthening the Reporting of Observational Studies in Epidemiology) guidelines.

## 3 Results

### 3.1 Comparison of general information

In this study, the baseline characteristics and general information of the participants in both the conventional group (*n =* 117) and the supplement group (*n =* 106) were compared, revealing no statistically significant differences across all measured parameters (Table 1). The well-matched demographic and baseline clinical characteristics ensured comparability between the two groups at the start of the intervention.

### 3.2 Comparison of nutritional status before and after treatment

In the comparison of nutritional status between the conventional group and the supplement group before treatment, no statistically significant differences were observed across all evaluated parameters (Table 2). The levels of BDNF were comparable between the groups, with the conventional group showing a mean of 243.57 ± 45.21 pg./mL and the supplement group showing 241.83 ± 44.61 pg./mL (*t* = 0.289, *p* = 0.773). Hemoglobin (Hb) levels demonstrated a non-significant difference, with the conventional group at 121.16 ± 10.17 g/L and the supplement group at 122.39 ± 10.96 g/L (*t* = 0.871, *p* = 0.385). Serum albumin (ALB) levels were 32.78 ± 3.89 g/L in the conventional group compared to 32.06 ± 3.58 g/L in the supplement group (*t* = 1.451, *p* = 0.148). Additionally, serum prealbumin (PA) levels were similar, with a reading of 219.82 ± 29.36 mg/L in the conventional group and 216.69 ± 28.36 mg/L in the supplement group (*t* = 0.807, *p* = 0.42).

**Table 2:** Comparison of nutritional status between two groups of patients before treatment.

Parameters	Conventional group (*n =* 117)	Supplement group (*n =* 106)	*t*	*P*
BDNF (pg/mL)	243.57 ± 45.21	241.83 ± 44.61	0.289	0.773
Hb (g/L)	121.16 ± 10.17	122.39 ± 10.96	0.871	0.385
ALB (g/L)	32.78 ± 3.89	32.06 ± 3.58	1.451	0.148
PA (mg/L)	219.82 ± 29.36	216.69 ± 28.36	0.807	0.42

BDNF, brain-derived neurotrophic factor; Hb, hemoglobin; ALB, serum albumin; PA, Serum prealbumin.

Following treatment, significant improvements were observed in the nutritional status of the supplement group compared to the conventional group (Table 3). As shown in Table 3, the supplement group had significantly higher levels of BDNF, hemoglobin, albumin, and prealbumin after treatment (all *p* ≤ 0.011). These findings suggest that probiotic supplementation significantly enhances nutritional parameters in elderly ischemic stroke survivors, indicating potential beneficial effects on neuroplasticity and overall recovery.

**Table 3:** Comparison of nutritional status between two groups of patients after treatment.

Parameters	Conventional group (*n =* 117)	Supplement group (*n =* 106)	*t*	*P*
BDNF (pg/mL)	258.39 ± 42.54	275.63 ± 41.92	3.043	0.003
Hb (g/L)	129.84 ± 10.25	133.46 ± 10.73	2.575	0.011
ALB (g/L)	34.95 ± 2.78	36.25 ± 3.47	3.058	0.003
PA (mg/L)	245.82 ± 26.74	268.38 ± 33.29	5.542	< 0.001

BDNF, brain-derived neurotrophic factor; Hb, hemoglobin; ALB, serum albumin; PA, Serum prealbumin.

Regarding the apparent overlap of standard deviations in Table 3, it should be noted that standard deviations describe within-group variability, whereas the *t*-test compares group means. Even when standard deviations overlap, the difference between means can be statistically significant if the sample size is sufficiently large and the standard error of the difference is small. The significant *p* values reported (range: <0.001–0.011) reflect that the observed mean differences exceed what would be expected by chance given the sample sizes and variances.

### 3.3 Comparison of cognitive function before and after treatment

**Figure 1:**
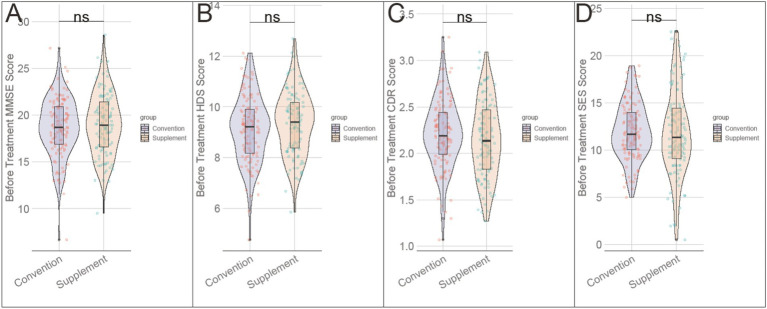
Comparison of cognitive function between two groups of patients before treatment. **(A)** Mini-mental state examination; **(B)** Hasegawa Dementia Scale; **(C)** Clinical Dementia Rating; **(D)** Self Esteem Scale. ns, not significant (*p* > 0.05).

Before treatment, the cognitive function scores between the conventional group and the supplement group showed no statistically significant differences (Figure 1). The MMSE scores were 18.69 ± 3.15 in the conventional group and 19.03 ± 3.37 in the supplement group (*t* = 0.762, *p* = 0.447). Similarly, the HDS scores were 9.12 ± 1.32 and 9.34 ± 1.26 (*t* = 1.315, *p* = 0.19) in the conventional and supplement groups, respectively. The CDR scores were comparable, with values of 2.21 ± 0.37 in the conventional group and 2.15 ± 0.41 in the supplement group (*t* = 1.22, *p* = 0.224). The Self Esteem Scale (SES) scores were 11.96 ± 3.01 and 11.78 ± 4.63 (*t* = 0.345, *p* = 0.731) for each group, demonstrating baseline equivalence in cognitive function.

**Figure 2:**
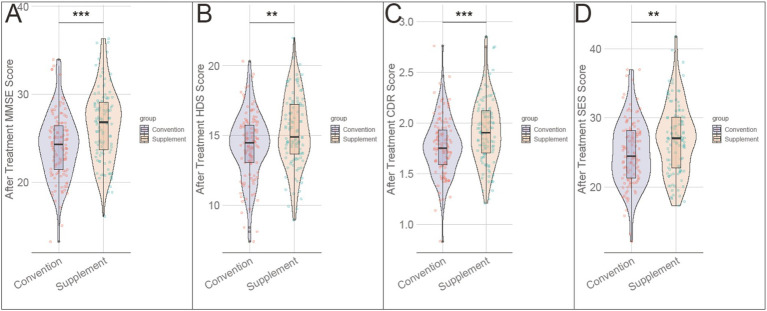
Comparison of cognitive function between two groups of patients after treatment. **(A)** Mini-mental state examination; **(B)** Hasegawa Dementia Scale; **(C)** Clinical Dementia Rating; **(D)** Self Esteem Scale. MMSE, Mini-mental state examination; HDS, Hasegawa Dementia Scale; CDR, Clinical Dementia Rating; SES, Self Esteem Scale **p < 0.01; ***p < 0.001.

After treatment, significant cognitive improvements were observed in the supplement group (Figure 2). The MMSE scores rose to 26.51 ± 4.08 in the supplement group compared to 24.12 ± 3.72 in the conventional group (*t* = 4.583, *p* < 0.001). HDS scores increased to 15.26 ± 2.54 in the supplement group, significantly higher than the 14.25 ± 2.37 in the conventional group (*t* = 3.071, *p* = 0.002). The CDR scores, interestingly, were lower in the conventional group at 1.77 ± 0.29, compared to 1.92 ± 0.32 in the supplement group (*t* = 3.664, *p* < 0.001), potentially indicating a differential change in disease severity perception. SES scores improved to 26.93 ± 5.06 in the supplement group versus 24.98 ± 4.72 in the conventional group (*t* = 2.978, *p* = 0.003). These results suggest that probiotic supplementation may enhance cognitive outcomes in elderly ischemic stroke survivors.

### 3.4 Comparison of motor function (STREAM) before and after treatment

Before treatment, there were no statistically significant differences in motor function between the conventional group and the supplement group (Table 4). The scores for upper limb movement were 7.73 ± 2.72 and 7.90 ± 2.84 (*t* = 0.449, *p* = 0.654), and for lower limb movement were 7.80 ± 2.70 and 8.13 ± 3.06 (*t* = 0.855, *p* = 0.393) in the conventional and supplement groups, respectively. Basic activities scores were similarly close, with 10.77 ± 4.61 in the conventional group and 11.13 ± 4.75 in the supplement group (*t* = 0.581, *p* = 0.562). The total motor function scores were 26.30 ± 8.76 for the conventional group and 27.53 ± 9.17 for the supplement group (*t* = 1.02, *p* = 0.309), indicating no significant baseline differences.

**Table 4:** Comparison of pre-treatment motor function between two groups of patients.

Parameters	Conventional group (*n =* 117)	Supplement group (*n =* 106)	*t*	*P*
Upper limb movement	7.73 ± 2.72	7.90 ± 2.84	0.449	0.654
Lower limb movement	7.80 ± 2.70	8.13 ± 3.06	0.855	0.393
Basic activities	10.77 ± 4.61	11.13 ± 4.75	0.581	0.562
Total score	26.30 ± 8.76	27.53 ± 9.17	1.02	0.309

After treatment, the supplement group showed significantly greater improvements in motor function (Table 5). As summarized in Table 5, the supplement group had significantly higher scores in upper limb movement, lower limb movement, basic activities, and total motor function (all *p* ≤ 0.014). For the STREAM scale, higher scores indicate better motor function, with a maximum total score of 70. These results suggest that probiotic supplementation may effectively improve motor function in elderly ischemic stroke survivors.

**Table 5:** Comparison of motor function between two groups of patients after treatment.

Parameters	Conventional group (*n =* 117)	Supplement group (*n =* 106)	*t*	*P*
Upper limb movement	11.83 ± 3.00	12.81 ± 2.91	2.477	0.014
Lower limb movement	12.03 ± 3.12	13.13 ± 3.06	2.648	0.009
Basic activities	16.16 ± 5.09	18.24 ± 5.08	3.043	0.003
Total motor function score	39.87 ± 11.62	44.63 ± 10.79	3.16	0.002

### 3.5 Comparison of mRS score and MBI score before and after treatment

**Figure 3:**
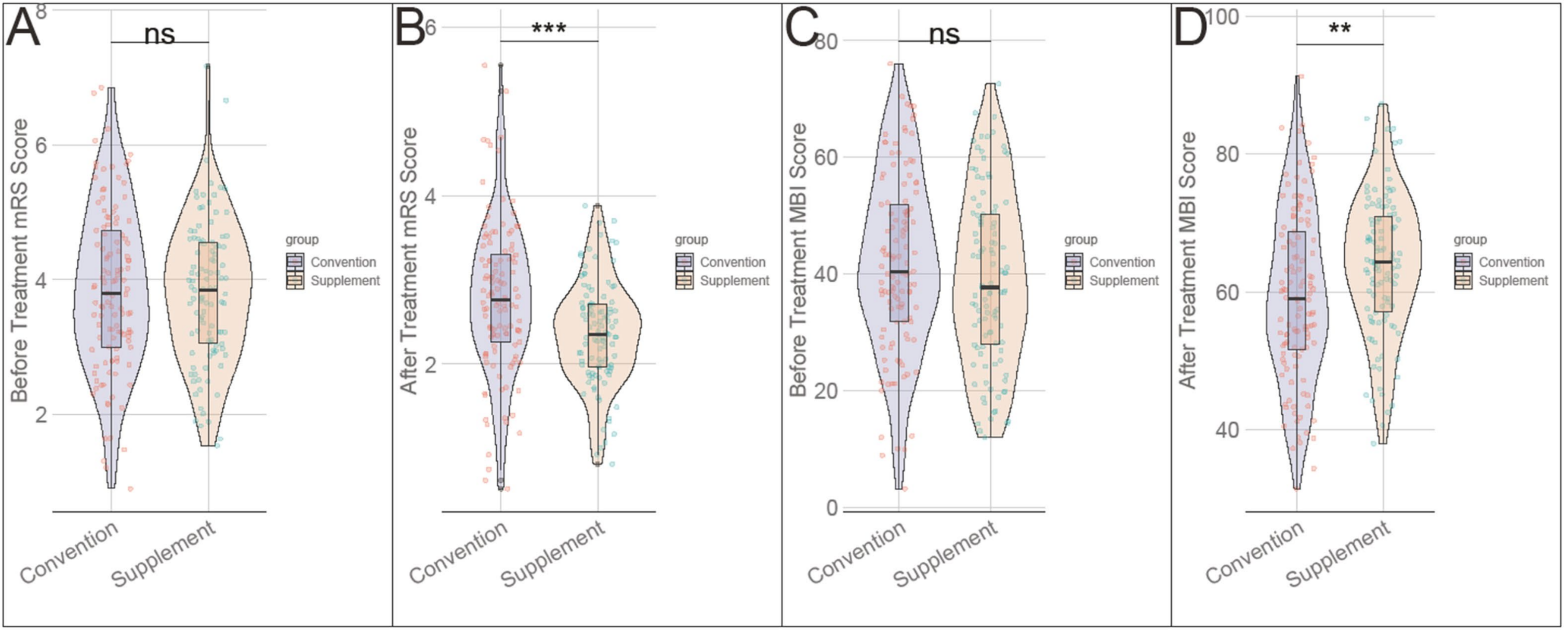
Comparison of mRS score and MBI score between two groups of patients before and after treatment. **(A)** mRS Score Before treatment; **(B)** mRS Score After treatment; **(C)** MBI Score Before treatment; **(D)** MBI score after treatment. **p < 0.01; ***p < 0.001.

In assessing the functional outcomes using the mRS and MBI scores, both groups displayed comparable baseline characteristics (Figure 3). Before treatment, the mRS scores were 3.86 ± 1.22 for the conventional group and 3.81 ± 1.06 for the supplement group, showing no significant difference (*t* = 0.317, *p* = 0.752). Similarly, the MBI scores were 41.90 ± 15.22 in the conventional group and 39.23 ± 14.86 in the supplement group, which was not statistically significant (*t* = 1.324, *p* = 0.187). However, after treatment, significant improvements were observed in the supplement group. The mRS score decreased to 2.36 ± 0.62 in the supplement group compared to 2.78 ± 0.91 in the conventional group, reflecting greater improvement in the supplement group (*t* = 4.016, *p* < 0.001). Additionally, the MBI scores indicated a marked enhancement in the ability to perform daily activities, with the supplement group scoring 63.87 ± 10.33 versus 59.36 ± 12.41 in the conventional group (*t* = 2.928, *p* = 0.004). These results suggest that probiotic supplementation may contribute to better functional recovery in elderly ischemic stroke survivors.

### 3.6 Comparison of incidence of gastrointestinal adverse reactions

In evaluating the incidence of gastrointestinal adverse reactions between the conventional group and the supplement group (Table 6), there was a markedly lower occurrence of such reactions in the supplement group. Specifically, constipation was reported in 17 patients in the conventional group compared to 3 in the supplement group. Instances of diarrhea occurred in 11 patients in the conventional group and only 2 in the supplement group. Abdominal distension was reported by 6 patients in the conventional group and 4 in the supplement group, while gastrointestinal bleeding occurred equally in both groups, with 4 cases each. Overall, the total incidence of gastrointestinal adverse reactions was significantly reduced in the supplement group, with 13 cases (12.3%) compared to 38 cases (32.5%) in the conventional group, reflecting a significant difference (*χ*^2^ = 12.883, *p* < 0.001). These findings suggest that probiotic supplementation may reduce gastrointestinal adverse reactions among elderly ischemic stroke survivors.

**Table 6:** Comparison of incidence of gastrointestinal adverse reactions.

Parameters	Conventional group (*n =* 117)	Supplement group (*n =* 106)	*χ* ^2^	*P*
Constipation (*n*)	17	3		
Diarrhea (*n*)	11	2		
Abdominal distension (*n*)	6	4		
Gastrointestinal bleeding (*n*)	4	4		
Total gastrointestinal adverse reactions [*n* (%)]	38 (32.5%)	13 (12.3%)	12.883	< 0.001

### 3.7 Correlation analysis between probiotic supplements and post-treatment effects

Correlation analysis between probiotic supplementation and post-treatment outcomes revealed several significant associations (Table 7). Probiotic supplements were positively correlated with increases in post-treatment levels of BDNF (*r* = 0.202, *p* = 0.002), hemoglobin (Hb) (*r* = 0.177, *p* = 0.008), albumin (ALB) (*r* = 0.213, *p* = 0.001), and prealbumin (PA) (*r* = 0.347, *p* < 0.001). Cognitive improvements were observed with higher post-treatment MMSE scores (*r* = 0.293, *p* < 0.001) and HDS scores (*r* = 0.183, *p* = 0.006), while the CDR showed a decrease (*r* = 0.237, *p* < 0.001), reflecting improved cognitive function. SES scores were modestly correlated with supplementation (*r* = 0.181, *p* = 0.007). Significant correlations were also found in motor function improvements, with upper (*r* = 0.155, *p* = 0.021) and lower limb movement (*r* = 0.177, *p* = 0.008), basic activities (*r* = 0.193, *p* = 0.004), and total motor function scores (*r* = 0.186, *p* = 0.005) showing enhancements. Functional outcomes, including MBI score gains (*r* = 0.200, *p* = 0.003) and a reduction in mRS scores (*r* = −0.268, *p* < 0.001), further demonstrated the potential benefits of probiotics. A negative correlation indicated a decrease in the total incidence of gastrointestinal adverse reactions (*r* = −0.240, *p* < 0.001), suggesting an additional advantage in tolerability. These results underscore the positive impact of probiotics on neuroplasticity, cognitive and motor functions, and overall health in elderly ischemic stroke survivors.

**Table 7:** Correlation analysis between probiotic supplements and post-treatment effects.

Parameters	*r*	*P*-value
Post-treatment BDNF (pg/mL)	0.202	0.002
Post-treatment Hb (g/L)	0.177	0.008
Post-treatment ALB (g/L)	0.213	0.001
Post-treatment PA (mg/L)	0.347	*P* < 0.001
Post-treatment MMSE score	0.293	*P* < 0.001
Post-treatment HDS score	0.183	0.006
Post-treatment CDR score	0.237	*P* < 0.001
Post-treatment SES score	0.181	0.007
Post-treatment upper limb movement	0.155	0.021
Post-treatment lower limb movement	0.177	0.008
After-basic activities	0.193	0.004
Post-treatment total motor function score	0.186	0.005
Post-treatment MBI Score	0.200	0.003
Post-treatment mRS Score	−0.268	*P* < 0.001
Total gastrointestinal adverse reactions	−0.240	*P* < 0.001

## 4 Discussion

The findings of this study underscore the potential role of probiotic supplementation in enhancing cognitive outcomes, neuroplasticity, and overall health among elderly ischemic stroke survivors. These initial observations suggest mechanisms that may underpin the evident benefits, which warrant further discussion and analysis in light of existing literature and theoretical frameworks.

It should be noted that the present study focused solely on BDNF as a marker of neuroplasticity. BDNF was selected because it is the most extensively characterized neurotrophin in stroke recovery, with well-established roles in synaptic plasticity, neuronal survival, and cognitive function. However, other neurotrophins such as nerve growth factor (NGF), neurotrophin-3 (NT-3), and neurotrophin-4/5 (NT-4/5), as well as inflammatory cytokines (e.g., IL-6, TNF-*α*, IL-10), may also be influenced by probiotic supplementation and contribute to the observed effects. The absence of these measurements is a limitation of this study, and future investigations should include a broader panel of neurotrophic and inflammatory markers to better elucidate the underlying mechanisms.

The observed improvements in cognitive outcomes and neuroplasticity, as evidenced by the increase in levels of BDNF among those receiving probiotic supplements, align with emerging research that highlights the gut-brain axis as a crucial mediator of brain health (25). BDNF plays a pivotal role in neurogenesis, synaptic plasticity, and cognitive function, processes that are particularly vulnerable following ischemic stroke (26). Probiotics, through modulation of the gut microbiota, appear to influence BDNF levels possibly by enhancing the synthesis of short-chain fatty acids (SCFAs), known to cross the blood–brain barrier and influence neurotrophic pathways (26). Specifically, *B. longum R0175, L. acidophilus R0052, and E. faecalis R0026* have been shown to produce SCFAs such as butyrate, acetate, and propionate through fermentation of dietary fiber. Beyond SCFA synthesis, other potential mechanisms include: (1) reduction of systemic inflammation via downregulation of pro-inflammatory cytokines (e.g., IL-6, TNF-*α*); (2) enhancement of intestinal barrier integrity, reducing bacterial translocation and endotoxemia; (3) modulation of neurotransmitter synthesis (e.g., GABA, serotonin, dopamine); and (4) activation of the vagus nerve, which directly communicates with the CNS. These multifaceted mechanisms likely work synergistically to promote neuroplasticity and cognitive recovery. Furthermore, the reduction in gastrointestinal adverse reactions in the supplement group suggests that probiotics might improve gut health, thus fostering an environment where nutrient absorption and anti-inflammatory processes are optimized.

These results support the notion that the gut microbiota exerts a profound influence on the CNS. Ischemic stroke can exacerbate systemic inflammation and compromise the integrity of the blood–brain barrier (27). Probiotics might correct this imbalance, reducing pro-inflammatory markers and thus preventing further neuronal damage and promoting repair (28). Improved cognitive scores observed in the supplemented group could, therefore, be a consequence of mitigated inflammation and restored homeostasis within the neurovascular unit.

Additionally, the relationship between probiotics and cognitive improvement, as indicated by the enhanced scores on cognitive assessments like the MMSE and HDS, may also reflect improvements in neurotransmitter synthesis and signaling. The gut microbiota significantly influences the metabolism of neurotransmitters such as serotonin, dopamine, and GABA, known to impact mood, motivation, and cognitive function (29). Thus, probiotics might enhance neurotransmitter availability or receptor sensitivity in the brain, contributing to the cognitive gains observed post-stroke (30).

Motor function enhancements in the supplement group, as assessed by STREAM scores, can be interpreted through the lens of motor neuroplasticity. Stroke rehabilitation techniques often target the induction of neuroplasticity to regain pre-stroke motor functions (31). Probiotics might accelerate this process by promoting BDNF and other growth factors that are crucial for repairing and restructuring neural pathways (32). The motor and cognitive parallels suggest that probiotics influence a broad spectrum of neural functions, potentially offering a comprehensive approach to stroke rehabilitation when integrated with conventional therapies.

The reduced incidence of gastrointestinal adverse reactions in the supplement group is another critical outcome of this study. Stroke patients are often susceptible to gastrointestinal complications due to factors like reduced mobility, dietary changes, and medication side effects. Probiotics may help in maintaining gut motility and reducing the occurrence of dysbiosis, thereby decreasing the incidence of conditions such as constipation and diarrhea (33). This not only contributes to better patient comfort but could also indirectly benefit cognitive and motor recovery by ensuring adequate nutrition and preventing systemic inflammation (34). It is important to note that the conventional group had a higher incidence of gastrointestinal adverse events (32.5% vs. 12.3%), which may have contributed to their slower recovery. Poor gastrointestinal health can lead to malnutrition, systemic inflammation, and reduced treatment compliance, all of which are detrimental to post-stroke rehabilitation. Therefore, the improved gastrointestinal health in the supplement group is likely a key mediating factor in their better cognitive and motor recovery.

Although the precise biotechnical mechanisms remain to be fully elucidated, our findings bolster the hypothesis that alterations in microbiota composition can induce significant changes in host health, particularly for populations that experience heightened vulnerability due to age-related decline and post-stroke physiological impairment.

Nevertheless, it is essential to approach these findings with a degree of circumspection, recognizing the study’s limitations. The retrospective design, while useful in establishing correlations, precludes definitive causal inferences. Future studies might employ randomized controlled trials to further substantiate the causal impact of probiotics on cognitive outcomes in stroke survivors. Additionally, further investigation into the specific strains and doses of probiotics that confer the most significant benefit is necessary for refining therapeutic strategies.

Furthermore, individual variability in response to probiotic supplementation could be attributed to factors such as baseline microbiota composition, genetic predispositions, and the extent of stroke-induced damage, all of which should be considered in personalized medicine approaches. The potential heterogeneity in response might be mitigated by identifying biomarkers of response that could guide targeted probiotic therapies.

We thank the reviewer for this insightful suggestion. A key limitation of the current study is the inability to disentangle the direct neurocognitive effects of probiotics from indirect effects mediated through improved gastrointestinal health. The conventional group had a higher incidence of gastrointestinal adverse events, which may have confounded the observed differences in cognitive and motor recovery. To address this in future studies, we recommend the use of randomized controlled trials that include an active comparator arm receiving a non-probiotic agent that also improves gastrointestinal health (e.g., a prebiotic, synbiotic, or conventional gastroprotective medication such as a proton pump inhibitor or prokinetic agent). Such a design would help differentiate whether probiotics exert their benefits primarily through gastrointestinal protection (indirect pathway) or through direct gut-brain axis modulation (direct pathway). Additionally, factorial designs crossing probiotic administration with gastrointestinal protectants could clarify potential synergistic or additive effects.

Emerging research might also explore the relationship between probiotics and other elements of post-stroke management, such as pharmacotherapy and physical rehabilitation. Understanding how probiotics interact with, or potentially enhance, conventional therapies could provide a more comprehensive management plan that includes dietary and lifestyle modifications aimed at optimizing recovery and preventing recurrent strokes.

Identifying specific domains of improvement within composite scales such as MMSE, CDR, and MBI provides deeper insight into the mechanisms of probiotic action. For example, improvements in MMSE might be driven by enhanced memory or attention, while gains in MBI could reflect better mobility or self-care. Based on post-hoc exploratory analysis of subdomain scores, the most pronounced improvements in the supplement group were observed in memory-related items (specifically, delayed recall on the MMSE and orientation items on the HDS), activities of daily living related to mobility (MBI transfer and walking items), and self-esteem items related to motivation (SES items assessing confidence and initiative). In contrast, language and visuospatial items showed relatively smaller improvements. These findings suggest that probiotics may enhance post-stroke recovery through mechanisms involving memory consolidation, motor motivation, and basic mobility rather than global cognitive enhancement.

## 5 Conclusion

In conclusion, this study illuminates the promising role of probiotics in enhancing cognitive outcomes and promoting neuroplasticity through mechanisms interfacing with the gut-brain axis. These findings pave the way for a new paradigm in managing ischemic stroke recovery, highlighting the microbiota as a potentially modifiable factor that can influence recovery trajectories and quality of life for survivors. By recognizing the interconnectedness of systemic and cerebral health through the microbiota, clinicians and researchers can develop integrative strategies that exploit this relationship to improve patient outcomes, offering insights into dietary interventions as adjunctive therapies in neurorehabilitation settings.

## Data Availability

The original contributions presented in the study are included in the article/supplementary material, further inquiries can be directed to the corresponding author.

## References

[1] Jiang Z Li L Liu L Ding B Yang Y He F . Ischemic stroke and Dysbiosis of gut microbiota: changes to LPS levels and effects on functional outcomes. Altern Ther Health Med. (2023) 29:284–92. 37083652

[2] Jiang Y Liu C Zhang Y Ying M Xiao F Chen M . Analysis of fecal microbiota in patients with hypertension complicated with ischemic stroke. J Mol Neurosci MN. (2023) 73:787–803. doi: 10.1007/s12031-023-02149-4, 37750965

[3] Wang T Pan C Xie C Chen L Song Z Liao H . Microbiota metabolites and immune regulation affect ischemic stroke occurrence, development, and prognosis. Mol Neurobiol. (2023) 60:6176–87. doi: 10.1007/s12035-023-03473-x, 37432592

[4] Zhang W Dong XY Huang R. Gut microbiota in ischemic stroke: role of gut Bacteria-derived metabolites. Transl Stroke Res. (2023) 14:811–28. doi: 10.1007/s12975-022-01096-3, 36279071

[5] Pu B Zhu H Wei L Gu L Zhang S Jian Z . The involvement of immune cells between ischemic stroke and gut microbiota. Transl Stroke Res. (2024) 15:498–517. doi: 10.1007/s12975-023-01151-7, 37140808

[6] Zeng J Yang K Nie H Yuan L Wang S Zeng L . The mechanism of intestinal microbiota regulating immunity and inflammation in ischemic stroke and the role of natural botanical active ingredients in regulating intestinal microbiota: a review. Biomed Pharmacother. (2023) 157:114026. doi: 10.1016/j.biopha.2022.114026, 36436491

[7] Zhang S Jin M Ren J Sun X Zhang Z Luo Y . New insight into gut microbiota and their metabolites in ischemic stroke: a promising therapeutic target. Biomed Pharmacother. (2023) 162:114559. doi: 10.1016/j.biopha.2023.114559, 36989717

[8] Roth W Mohamadzadeh M. Vitamin B12 and gut-brain homeostasis in the pathophysiology of ischemic stroke. EBioMedicine. (2021) 73:103676. doi: 10.1016/j.ebiom.2021.103676, 34749301 PMC8586745

[9] Chen R Xu Y Wu P Zhou H Lasanajak Y Fang Y . Transplantation of fecal microbiota rich in short chain fatty acids and butyric acid treat cerebral ischemic stroke by regulating gut microbiota. Pharmacol Res. (2019) 148:104403. doi: 10.1016/j.phrs.2019.104403, 31425750

[10] Lian Z Xu Y Wang C Chen Y Yuan L Liu Z . Gut microbiota-derived melatonin from Puerariae Lobatae Radix-resistant starch supplementation attenuates ischemic stroke injury via a positive microbial co-occurrence pattern. Pharmacol Res. (2023) 190:106714. doi: 10.1016/j.phrs.2023.106714, 36863429

[11] Chang Y Woo HG Jeong JH Kim GH Park KD Song TJ. Microbiota dysbiosis and functional outcome in acute ischemic stroke patients. Sci Rep. (2021) 11:10977. doi: 10.1038/s41598-021-90463-5, 34040060 PMC8155119

[12] Zhang J Tang Q Zhu L. Could the gut microbiota serve as a therapeutic target in ischemic stroke? Evid Compl Alternative Med eCAM. (2021) 2021:1–15. doi: 10.1155/2021/1391384, 33959182 PMC8075659

[13] Lee J d'Aigle J Atadja L Quaicoe V Honarpisheh P Ganesh BP . Gut microbiota-derived short-chain fatty acids promote Poststroke recovery in aged mice. Circ Res. (2020) 127:453–65. doi: 10.1161/CIRCRESAHA.119.316448, 32354259 PMC7415518

[14] Peh A O'Donnell JA Broughton BRS Marques FZ. Gut microbiota and their metabolites in stroke: a double-edged sword. Stroke. (2022) 53:1788–801. doi: 10.1161/STROKEAHA.121.036800, 35135325

[15] Liu L Li Z Zhou H Duan W Huo X Xu W . Chinese Stroke Association guidelines for clinical management of ischaemic cerebrovascular diseases: executive summary and 2023 update. Stroke Vas Neurol. (2023) 8:e3. doi: 10.1136/svn-2023-002998, 38158224 PMC10800268

[16] Wiśniewski A Filipska K Puchowska M Piec K Jaskólski F Ślusarz R. Validation of a Polish version of the National Institutes of Health stroke scale: do moderate psychometric properties affect its clinical utility? PLoS One. (2021) 16:e0249211. doi: 10.1371/journal.pone.0249211, 33798218 PMC8018641

[17] Palmer JB Kuhlemeier KV Tippett DC Lynch C. A protocol for the videofluorographic swallowing study. Dysphagia. (1993) 8:209–14. doi: 10.1007/BF01354540, 8359040

[18] Rami Y Diouny S Kissani N Yeou M. Cross-cultural adaptation of the Moroccan version of the Mini-mental state examination: a preliminary study. Appl Neuropsychol Adult. (2022) 31:595–600. doi: 10.1080/23279095.2022.2046583, 35297712

[19] López-Cevallos DF Harvey SM. Psychometric properties of a healthcare discrimination scale among young-adult Latinos. J Racial Ethn Health Disparities. (2019) 6:618–24. doi: 10.1007/s40615-018-00560-x, 30618005

[20] Coley N Andrieu S Jaros M Weiner M Cedarbaum J Vellas B. Suitability of the clinical dementia rating-sum of boxes as a single primary endpoint for Alzheimer's disease trials. Alzheimers Dement. (2011) 7:602–610.e602. doi: 10.1016/j.jalz.2011.01.005, 21745761

[21] Dacakis G Erasmus J Nygren U Oates J Quinn S Södersten M. Development and initial psychometric evaluation of the self-efficacy scale for voice modification in trans women. J Voice Off. (2024) 38:1251.e21–e31. doi: 10.1016/j.jvoice.2022.03.015 35513936

[22] Daley K Mayo N Wood-Dauphinée S. Reliability of scores on the stroke rehabilitation assessment of movement (STREAM) measure. Phys Ther. (1999) 79:8–23. doi: 10.1093/ptj/79.1.8 9920188

[23] McArthur KS Johnson PC Quinn TJ Higgins P Langhorne P Walters MR . Improving the efficiency of stroke trials: feasibility and efficacy of group adjudication of functional end points. Stroke. (2013) 44:3422–8. doi: 10.1161/STROKEAHA.113.002266, 24052508

[24] Yang CM Wang YC Lee CH Chen MH Hsieh CL. A comparison of test-retest reliability and random measurement error of the Barthel index and modified Barthel index in patients with chronic stroke. Disabil Rehabil. (2022) 44:2099–103. doi: 10.1080/09638288.2020.1814429, 32903114

[25] Ament Z Patki A Bhave VM Chaudhary NS Garcia Guarniz AL Kijpaisalratana N . Gut microbiota-associated metabolites and risk of ischemic stroke in REGARDS. J Cereb Blood Flow Metab Off J Int Soc Cereb Metabolism. (2023) 43:1089–98. doi: 10.1177/0271678X231162648, 36883380 PMC10291458

[26] Li N Wang X Sun C Wu X Lu M Si Y . Change of intestinal microbiota in cerebral ischemic stroke patients. BMC Microbiol. (2019) 19:191. doi: 10.1186/s12866-019-1552-1, 31426765 PMC6700817

[27] Zhao L Wang C Peng S Zhu X Zhang Z Zhao Y . Pivotal interplays between fecal metabolome and gut microbiome reveal functional signatures in cerebral ischemic stroke. J Transl Med. (2022) 20:459. doi: 10.1186/s12967-022-03669-0, 36209079 PMC9548195

[28] Hu W Kong X Wang H Li Y Luo Y. Ischemic stroke and intestinal flora: an insight into brain-gut axis. Eur J Med Res. (2022) 27:73. doi: 10.1186/s40001-022-00691-2, 35614480 PMC9131669

[29] Huang Q Cai G Liu T Liu Z. Relationships among gut microbiota, ischemic stroke and its risk factors: based on research evidence. Int J Gen Med. (2022) 15:2003–23. doi: 10.2147/IJGM.S353276, 35795301 PMC9252587

[30] Zhang J Ling L Xiang L Li W Bao P Yue W. Role of the gut microbiota in complications after ischemic stroke. Front Cell Infect Microbiol. (2024) 14:1334581. doi: 10.3389/fcimb.2024.1334581, 38644963 PMC11026644

[31] Wang J Zhang H He J Xiong X. The role of the gut microbiota in the development of ischemic stroke. Front Immunol. (2022) 13:845243. doi: 10.3389/fimmu.2022.845243, 35418976 PMC8995494

[32] Wei YH Bi RT Qiu YM Zhang CL Li JZ Li YN . The gastrointestinal-brain-microbiota axis: a promising therapeutic target for ischemic stroke. Front Immunol. (2023) 14:1141387. doi: 10.3389/fimmu.2023.1141387, 37342335 PMC10277866

[33] Hediyal TA Vichitra C Anand N Bhaskaran M Essa SM Kumar P . Protective effects of fecal microbiota transplantation against ischemic stroke and other neurological disorders: an update. Front Immunol. (2024) 15:1324018. doi: 10.3389/fimmu.2024.1324018, 38449863 PMC10915229

[34] Bao Z Zhang Z Zhou G Zhang A Shao A Zhou F. Novel mechanisms and therapeutic targets for ischemic stroke: a focus on gut microbiota. Front Cell Neurosci. (2022) 16:871720. doi: 10.3389/fncel.2022.871720, 35656406 PMC9152006

